# The Concept of Ecthyma Gangrenosum Illustrated by a *Fusarium oxysporum* Infection in an Immunocompetent Individual

**DOI:** 10.1007/s11046-016-0031-6

**Published:** 2016-06-21

**Authors:** Yanping Jiang, Abdullah M. S. Al-Hatmi, Yining Xiang, Yu Cao, Albert H. G. Gerrits van den Ende, Ilse Curfs-Breuker, Jacques F. Meis, Hongguang Lu, G. Sybren de Hoog

**Affiliations:** 1 Department of Dermatology, The Affiliated Hospital, Guizhou Medical University, PO Box 550001, 4 Beijing Road, Guiyang, China; 2 CBS-KNAW Fungal Biodiversity Centre, PO Box 85167, 3508 AD Utrecht, The Netherlands; 3 Institute of Biodiversity and Ecosystem Dynamics, University of Amsterdam, Amsterdam, The Netherlands; 4 Directorate General of Health Services, Ibri Hospital, Ministry of Health, Muscat, Oman; 5 Departments of Pathology, The Affiliated Hospital, Guizhou Medical University, Guiyang, China; 6 Department of Medical Microbiology and Infectious Diseases, Canisius Wilhelmina Hospital, Nijmegen, The Netherlands; 7 Department of Medical Microbiology, Radboud University Medical Centre, Nijmegen, The Netherlands; 8 Basic Pathology Department, Federal University of Paraná State, Curitiba, Paraná Brazil; 9 Biology Department, Faculty of Science, King Abdulaziz University, Jeddah, Saudi Arabia

**Keywords:** *Fusarium oxysporum*, Ecthyma gangrenosum, Definition, Immunocompetent

## Abstract

Ecthyma gangrenosum (EG) involves necrotic cutaneous lesions caused by bacteria, mainly *Pseudomonas aeruginosa*, and is usually seen in immunocompromised patients with septicemia. However, clinically similar infections have been published with fungi as etiologic agents. We present a case of an EG-like lesion due to *Fusarium oxysporum* confirmed by clinical diagnosis, culture and molecular identification and discuss the definition of EG.

## Introduction

Ecthyma gangrenosum (EG) is generally defined as an infection with multiple necrotic cutaneous ulcers caused by species of *Pseudomonas*, mainly *P. aeruginosa*, or *Aeromonas.* The disorder is usually seen in immunocompromised patients, particularly those with underlying malignant disease. Occurrence in immunocompetent individuals is highly exceptional [1]. Since 1980s, data have accumulated on a wider bacterial spectrum of gram-negative agents like *Escherichia coli,Citrobacter freundii*, *Klebsiella pneumonia*, *Morganella morganii* and various *Pseudomonas* species [2, 3]. Also some fungi have been reported to cause clinically similar lesions, e.g., species of *Candida*, *Aspergillus*, and *Curvularia* [4]. Thus, the disease definition for some authors is determined by the etiologic agent and for others by clinical features. Nevertheless, the name is infrequently applied to fungal infections. This confusion may mask a wider prevalence of EG-like infections, which otherwise may have been reported under a wide spectrum of fungal diseases. We present a fungal case of an EG-like infection in an immunocompetent patient caused by *Fusarium oxysporum* and discuss the definition of EG.

## Case Presentation

**Fig. 1:**
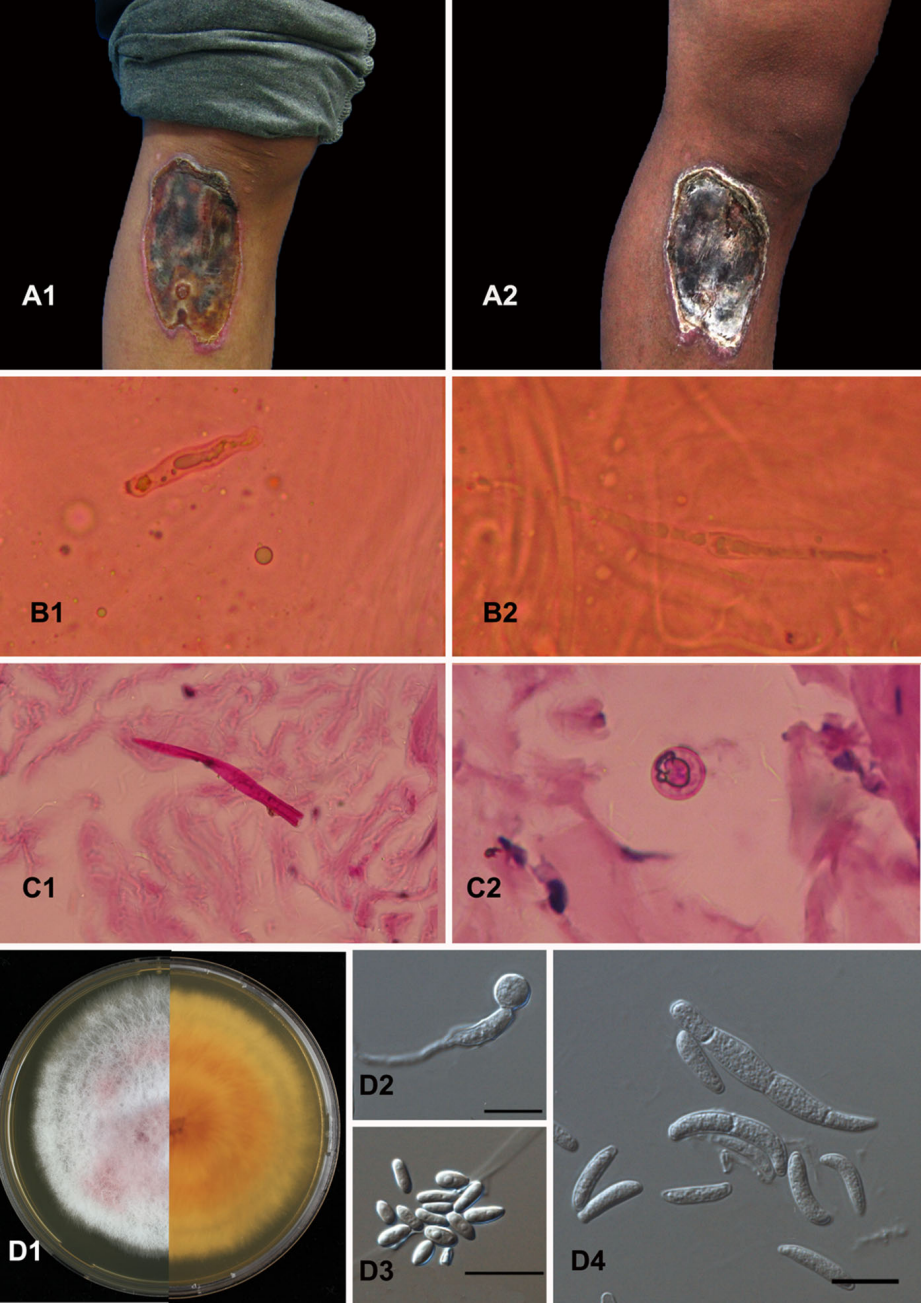
**a1** Initial infection; **a2** After 2 weeks of treatment; **b1**, **b2** KOH mount of the swab collected from the lesion showing fungal hyaline hyphae (magnification, ×40); **c1**, **c2** fungal hyphae seen with H&E stain from the biopsy; **d1** growth of white colonies and purple pigmentation on MEA at 25 °C.; **d2**–**d4** septate macroconidia and microconidia. All *scale bars* 10 μm

A 17-year-old male from southwestern China with epilepsy presented with a painless ulcer on his right leg which was clinically suspected as being dry gangrene. The lesion started 2 months earlier when he was injured during an epileptic insult by a wooden bench. Initial lesions were in the form of small, painless papules which quickly progressed to a painless ulcer with a black eschar over a period of 2 weeks. All laboratory investigations were within normal ranges, and in particular, no immune deficiency was apparent. However, the patient had a history of epilepsy for which he was on carbamazepine for 7 years. Examination of the skin lesion showed a 5 × 13 cm skin ulcer with black eschar, i.e., the ulcer edge appeared to “roll over” and took the shape of a tough, erythematous and fibrous ring. The black eschar adhered tightly without discharge of pus or granules (a1, a2). Upon first visit, the case was diagnosed as EG (Fig. 1).

## Identification of the Fungal Etiological Agent

A skin biopsy was taken from the lesion and direct examination (KOH 10 %) showed multiple thin hyaline hyphae (b1, b2). Histopathological examination showed irregular, hyaline hyphae in the H&E-stained tissue (c1, c2). Culture on Sabouraud’s glucose agar (SGA) showed white colonies with a light brown pigment excreted into the medium. Phenotypic identification of this isolate resulted in a *Fusarium* species. Since fungal hyphae were visible in histopathological slides, the medical judgment of the case was changed from EG to an EG-like fungal infection. Subsequently, voriconazole (400 mg/day orally) was added to the therapeutic plan at day 14 after admission. Two weeks after the start of antifungal treatment, the patient received surgical debridement and continued on oral voriconazole for another 2 weeks with noticeable improvement. The patient did not return for follow-up visits. The isolate was preserved and deposited in the CBS-KNAW reference collection with accession number CBS 140424.

Subculturing of the isolate for a more thorough morphological study was performed on Malt Extract Agar (MEA) and Oatmeal Agar (OA) with incubation times of 1 week at 25, 33 and 37 °C. Rapidly growing, hairy, pink–violet colonies were observed after incubation on MEA after 5 days at 28 °C. Microscopic morphology showed short, single, lateral monophialides (flask-shaped projections) in the aerial mycelium. Macroconidia were fusiform, slightly curved, 1–3 septate, 21.4–38.5 µm in length and 3.2–4.5 µm in width. Microconidia were abundant, not arranged in chains, 0–1 septate, 6.4–14.3 µm in length and 2.8–4.3 µm in width (d1–d4). Optimal growth was observed at 27 °C; maximum growth temperature was 37 °C. On the basis of culture and microscopy, the fungus was provisionally identified as *F. oxysporum*.

Genomic DNA was extracted from a culture grown on MEA following a cetyltrimethyl ammonium bromide protocol as described previously [5]. The rDNA internal transcribed spacer region (ITS) was analyzed as routine marker using primers ITS1 and ITS4. Since ITS is not a recommended locus for reliable identification of *Fusarium* species, the partial translation elongation factor 1-alpha (TEF1) and the partial RNA polymerase second largest subunit (rPB2) were also analyzed. PCR amplification and sequencing of the partial fragments were, respectively, done for TEF1 with primers EF1 and EF2R and for RPB2 with primers RPB2-7cR and RPB2-5F2 [5]. To perform identification, a similarity search with the sequences of each gene was done using the BLAST tool of the NCBI database, the FUSARIUM-ID database and the *Fusarium* MLST database. These BLAST searches yielded 100 % similarity scores with several submissions under the name *F. oxysporum*. The ITS, TEF1 and rPB2 nucleotide sequences were deposited in GenBank with accession numbers KT794176, KT794174 and KT794173, respectively.

## Antifungal Susceptibility Testing

Antifungal susceptibility testing was performed according to CLSI M38A (Clinical and Laboratory Standards Institute 2008) method, slightly modified by Al-Hatmi et al. [6] and demonstrated that the fungus had a low minimum inhibitory concentrations (MICs) of 1 µg/ml against amphotericin B and high MICs above published epidemiological cut off values (ECVs) [7], for itraconazole (>16 µg/ml), voriconazole (8 µg/ml), posaconazole (>16 µg/ml), isavuconazole (8 µg/ml), anidulafungin (>16 µg/ml), micafungin (>16 µg/ml), and fluconazole (>64 µg/ml).

## Discussion

At present EG is generally defined as a condition pathognomonic for *Pseudomonas aeruginosa* or related bacteria in immunocompromised patients. In the current literature, a much wider definition is often maintained, including variable etiology and variable host factors, also including immunocompetent. In our view, the gangrenous clinical appearance is the unique feature of the infection and should determine the definition, as follows: (1) Pathogens, which may be bacteria but also fungi, yeasts or mycobacteria are isolated from blood cultures and skin biopsies [8], (2) The host is immunocompromised. (3) Clinically, the lesions demonstrate hemorrhagic pustules that lead to necrotic ulcers which evolve into gangrenes with black scab and in a later stage become surrounded by a red halo. (4) Histopathology shows a necrotic epidermis and papillary dermal edema; venules are congested and red blood cell extravasation is noted in the papillary dermis; secondary thrombosis of the arterioles, tissue edema and separation of the epidermis leads to the specific picture of EG.

Consequently, our case was interpreted as EG-like, with presence of fungal hyphae, but a difference was noted in the fact that our patient was an immunocompetent individual. Although fungal agents are mostly reported from immunocompromised patients with EG, an immunocompromised status was judged not necessary [8]. This makes the clinical appearance of local gangrene even less specific. While originally *Pseudomonas* bacteria were part of the definition, now also other agents including fungi such as the general opportunist genus *Fusarium* can be involved, and the immune status of the host also appears variable. For all these reasons, it seems to us that a definition of EG by medical aspects of clinical features, and histopathology is most appropriate.

Reported fungal opportunists of EG-like and EG infections are *Aspergillus fumigatus*, *Candida albicans*, *C. tropicalis, Curvularia* sp., *Exserohilum* sp., *Fusarium solani*, *Metarhizium anisopliae*, *Mucor pusillus*, *Scedosporium boydii* and *Neoscytalidium dimidiatum* [4]. To this list, *F. oxysporum* can be added. Histopathology showed that local invasion with fungal elements was present in epidermis and dermis. An extensive literature search revealed six cases since 1975 in neutropenic patients due to *F. solani* or other *Fusarium* species that were listed as EG [9–14]. Our patient, without known immune deficiency, had a history of trauma during which the fungus could have been implanted in the skin. The eschar was tightly attached to the ulcer, suggesting that fungal EG might be different from bacterial EG, which would favor reference to fungal infections as “EG-like.” Though, more cases have to be studied to confirm this clinical difference. In most EG and EG-like cases, the clinical picture is specific; differential diagnosis should consider warfarin-induced skin necrosis, cocaine-induced skin necrosis, calciphylaxis, septic emboli, loxoscelism, diabetic microangiopathy, disseminated intravascular coagulation, paraneoplastic extensive necrotizing vasculitis, pyoderma gangrenosum, livedoid vasculopathy, antineutrophil cytoplasmic antibody -associated vasculitis, cutaneous necrotizing vasculitis due to familial Mediterranean fever, and necrosis secondary to the use of vasoactive drugs [15, 16].

Treatment of our patient was only for 2 weeks with voriconazole, but the beginning of a curative effect was seen during this short period. The loftiness around the lesion became flatter and the surface of the eschar became dry. EG is an ominous sign in immunocompromised patients, especially with *P. aeruginosa*. However, dermatologists should be aware that other pathogens such as fungi can cause clinically indistinguishable lesions. In most cases, the cutaneous appearance constitutes an important physical clue to the clinical diagnosis as an EG or EG-like lesion, that is black scab and surrounded by a red halo. Given the fact that the etiologic agent can be of bacterial and of fungal nature, it is essential that the pathogen is identified.
